# Conformational analysis of 2,2-difluoroethylamine hydrochloride: double *gauche* effect

**DOI:** 10.3762/bjoc.10.84

**Published:** 2014-04-16

**Authors:** Josué M Silla, Claudimar J Duarte, Rodrigo A Cormanich, Roberto Rittner, Matheus P Freitas

**Affiliations:** 1 Department of Chemistry, Federal University of Lavras, P. O. Box 3037, 37200-000, Lavras, MG, Brazil; 2 Chemistry Institute, State University of Campinas, P. O. Box 6154, 13084-971, Campinas, SP, Brazil

**Keywords:** conformational analysis, 2,2-difluoroethylamine hydrochloride, gauche effect, hydrogen bonding

## Abstract

The *gauche* effect in fluorinated alkylammonium salts is well known and attributed either to an intramolecular hydrogen bond or to an electrostatic attraction between the positively charged nitrogen and the vicinal electronegative fluorine atom. This work reports the effect of adding a fluorine atom in 2-fluoroethylamine hydrochloride on the conformational isomerism of the resulting 2,2-difluoroethylamine chloride (**2**). The analysis was carried out using NMR coupling constants in D_2_O solution, in order to mimic the equilibrium conditions in a physiological medium, in the gas phase and in implicit water through theoretical calculations. Despite the presence of σ_CH_→σ*_CF_ and σ_CH_→σ*_CN_ interactions, which usually rule the hyperconjugative *gauche* effect in 1,2-disubstituted ethanes, the most important forces leading to the double *gauche* effect (^+^NH_3_ in the *gauche* relationship with both fluorine atoms) in **2** are the Lewis-type ones. Particularly, electrostatic interactions are operative even in water solution, where they should be significantly attenuated, whereas hyperconjugation and hydrogen bond have secondary importance.

## Introduction

The conformational isomerism of alkylamines devotes interest because intramolecular effects relative to hydrocarbon analogues are affected by the electronegativity of the nitrogen atom and by the basicity of the amino group. However, most drug like molecules based on this class of compounds are protonated to give ammonium salts. In some cases, there is a strong conformational shift toward the *gauche* orientation between nitrogen and the electronegative substituent (such as the fluorine atom) after protonation of the nitrogen atom in a 2-substituted ethylamine fragment [1–5]. According to theoretical calculations, such conformational preference takes place in the gas phase and persists in water solution, where most biochemical processes occur.

We have recently shown that, in water solution, the *axial* preference of 3-fluoropiperidinium hydrochloride (F and N with *gauche* arrangement) is dependent on hyperconjugation, and not only due to F···HN^+^ hydrogen bond and/or electrostatic attraction between the electronegative fluorine with the positively charged nitrogen [6]. However, introduction of an additional 2-fluorine atom in that fragment to give 2,2-difluoroethylammonium salts generates incremental interactions and, thus, the contributions from Lewis and non-Lewis-type interactions should differ from those found in singly fluorinated ethylammonium salts.

The Lewis-type interactions result from four-electron/two-orbital interactions, such as steric effects and dipolar (electrostatic) interactions. On the other hand, non-Lewis-type interactions refer to electron delocalization from filled to empty orbitals, such as hyperconjugation. Indeed, the σ_CH_→σ*_CF_ hyperconjugative interaction has been found to be the main factor controlling the *gauche* effect in 1,2-difluoroethane and derivatives [7–9]. Nevertheless, the electrostatic *gauche* effect has been found to be operative in some β-fluoro-*N*-ethylpyridinium cations of interest, as well as in the C2'-*endo* conformation of NAD^+^ [5].

Since multifluorination represents a relevant challenge in organic synthesis and in the development of polar organic compounds with attractive properties [10] and because alkylammonium salts are present in a variety of pharmaceuticals, the present work focuses on describing the conformational isomerism in 2,2-difluoroethylamine hydrochloride.

## Results and Discussion

The conformational isomerism of 2,2-difluoroethylamine (**1**) was computationally investigated at the MP2/6-311++g(d,p) level, both in the gas phase and implicit water (using the Polarizable Continuum Model). The conformational preferences are consistent with those obtained elsewhere through theoretical calculations and infrared spectroscopy [11]. No significant double *gauche* effect has been found in **1**, since conformers possessing two fluorine atoms in the *gauche* relationship with the amino group (*gauche-gauche*, *gg*) are estimated to be similarly populated to those conformers with only one single fluorine *gauche* to the nitrogen atom (*anti-gauche*, *ag*) (Table 1). In fact, the most stable conformer of the neutral compound contains two fluorines *gauche* to the amino group, which presents both hydrogens directed toward fluorines, suggesting the formation of F···HN hydrogen bond. However, the second most stable form *ag* is calculated to be almost similar in energy with the global minimum, indicating that other intramolecular effects take place and/or that the above mentioned hydrogen bond makes a small contribution towards the stabilization of the global minimum. Second-order perturbation analysis of donor-acceptor interactions in the natural bond orbitals (NBOs) framework shows that the global minimum of **1** is more stabilized by hyperconjugation than the other conformers (both in the gas phase and implicit water), despite being significantly destabilized by Lewis-type interactions.

**Table 1:** Calculated parameters (in kcal mol^−1^) obtained for **1** in the gas phase and implicit water (conformer populations are given in parenthesis). Relative energies were obtained at the MP2/6-311++g(d,p) level and NBO data at the B3LYP/6-311++g(d,p) level.

	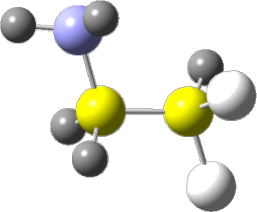	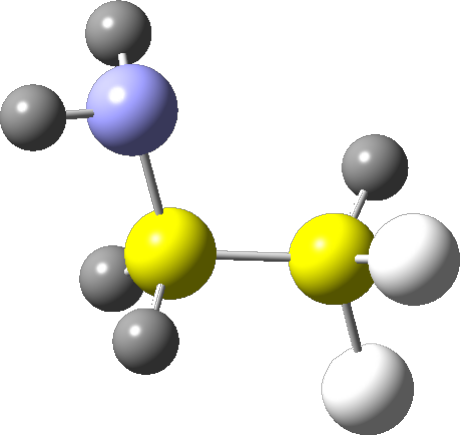	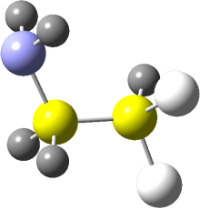	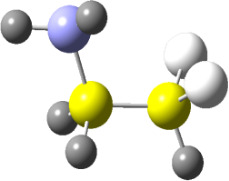	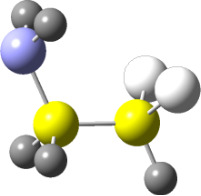
Parameter	*ag1*	*ag2*	*ag3*	*gg1*	*gg2*

*E*_rel (gas)_	0.2 (35%)	–	0.7 (15%)	2.4 (1%)	0.0 (49%)
*E*_Lewis (gas)_	0.2	–	7.7	5.3	7.3
*E*_hyperc (gas)_	0.0	–	−7.0	−2.9	−7.3
*E*_rel (water)_	0.4 (20%)	1.3 (4%)	0.3 (23%)	1.0 (14%)	0.0 (39%)
*E*_Lewis (water)_	3.6	1.3	7.4	3.6	8.6
*E*_hyperc (water)_	−3.2	0.0	−7.1	−2.6	−8.6

The conformational preference dramatically changes after protonation of **1** to give the 2,2-difluoroethylammonium cation (**2**), i.e*.* the conformer containing both fluorines *gauche* to the ammonium group (*gg*) is practically the single form in the equilibrium in the gas phase and, even in water solution, this conformer is calculated to amount to 90%. This preference is corroborated by NMR experiments (Supporting Information, File 1), because the measured ^3^*J*_H,H_ and ^3^*J*_H,F_ coupling constants (which have angular dependence according to the well known Karplus curve [12–14]) are consistent with the average values calculated for the *gg* conformer (Table 2). The experimental coupling constants for **2** in D_2_O solution are ^3^*J*_H,H_ = 2.6 Hz and ^3^*J*_H,F_ = 16.4 Hz, and the mean calculated values for the *gg* conformer in implicit water are 1.3 Hz and 16.8 Hz [(2.8 + 30.7)/2 = 16.8], while the corresponding values for the *ag* conformer are 5.5 Hz and 12.4 Hz.

**Table 2:** Calculated parameters (*E* in kcal mol^−1^ and *J* in Hz) obtained for **2** in the gas phase and implicit water (conformer populations are given in parenthesis).

Parameter	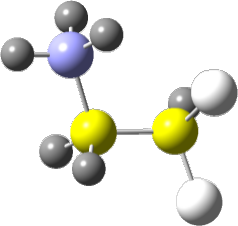 *ag*	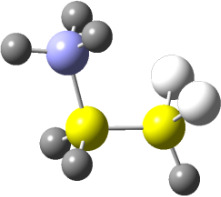 *gg*

*E*_rel (gas)_	4.2 (0%)	0.0 (100%)
*E*_Lewis (gas)_	12.4	0.0
*E*_hyperc (gas)_	−8.2	0.0
σ_CH_→σ*_CF (gas)_	2.7	3.0/3.0
σ_CH_→σ*_CN (gas)_		3.2
σ_CH_→σ*_CH (gas)_	2.0/1.7	
σ_CF_→σ*_CH (gas)_	0.8	0.9/0.9
σ_CN_→σ*_CH (gas)_		0.9
σ_CF_→σ*_CN (gas)_	1.4	
σ_CN_→σ*_CF (gas)_	1.1	
^3^*J*_H,H (gas)_	3.0/8.3	1.0/1.0
^3^*J*_H,F (gas)_	−1.9/5.3/9.4/23.9	2.3/2.3/24.0/24.0
*E*_rel (water)_	1.3 (10%)	0.0 (90%)
*E*_Lewis (water)_	1.8	0.0
*E*_hyperc (water)_	−0.5	0.0
σ_CH_→σ*_CF (water)_	3.5	3.7/3.7
σ_CH_→σ*_CN (water)_		3.4
σ_CH_→σ*_CH (water)_	2.2/1.9	
σ_CF_→σ*_CH (water)_	0.8	0.8/0.8
σ_CN_→σ*_CH (water)_		0.8
σ_CF_→σ*_CN (water)_	1.5	
σ_CN_→σ*_CF (water)_	1.3	
^3^*J*_H,H (water)_	2.2/8.8	1.3/1.3
^3^*J*_H,F (water)_	−0.7/7.4/10.4/32.3	2.8/2.8/30.7/30.7

**Figure 1:**
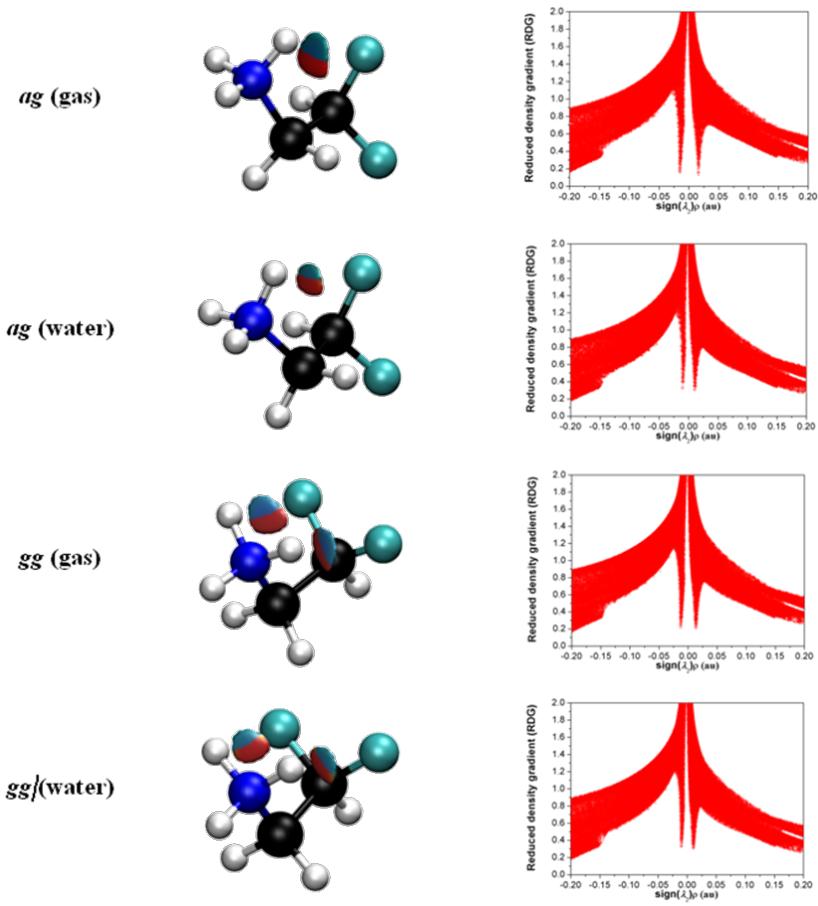
NCI domains and the plot of reduced density gradient (RDG) vs sign(*λ*_2_)*ρ* for the conformers of **2.** NCI isosurfaces were obtained with RDG = 0.5. The blue-red color scale ranges from −0.02 < sign(*λ*_2_)*ρ* < +0.02 au.

The positive charge on nitrogen attracts the fluorine atoms, while the F···HN^+^ hydrogen bond is not expected to be significantly affected if compared to **1**. In fact, QTAIM analysis does not capture any bond path between F and H(N^+^) to indicate a hydrogen bond. Likewise, the 'quantum' nature of this hydrogen bond (the *n*_F_→σ*_NH_ interaction) is not detected by NBO analysis. However, the new non-covalent interaction (NCI) approach, which is based on the electron density and its derivatives, enables the identification of non-covalent interactions by means of peaks that appear in the reduced density gradient (RDG) at low densities [15–17]. Indeed, the NCI method was capable of identifying F···HN^+^ hydrogen bond in **2** both in the gas phase and implicit water (Figure 1). For both **1** and **2**, NCI isosurfaces corresponding to F···HN^+^ hydrogen bonds are larger in the gas phase than in water solution and also the RDG values are closer to zero (Figure 1) for the first than the latter, indicating that such interactions are stronger in the gas phase than in water. The RDG peaks located in the negative valued sign(*λ*_2_)*ρ* graph area, which correspond to hydrogen bonds and refer to blue NCI isosurfaces in Figure 1, are 0.154 and 0.217 for the *ag* and *gg* geometries in the gas phase, respectively, while the corresponding values in water are higher (0.322 and 0.331). Also, the more negative −0.015 au and −0.013 au sign(*λ*_2_)*ρ* values for *ag* and *gg* in the gas phase in comparison with these conformers in water −0.010 au and −0.009 au, respectively, indicate that F···HN^+^ hydrogen bonds are stronger in the gas phase than in water from the NCI point of view. Since two interactions of this type are present in *gg* against only one in *ag*, the *gg* conformer is expected to be more stabilized by hydrogen bonds than *ag* conformer, even though the F···HN^+^ hydrogen bonds in *gg* are weaker than in *ag* as indicated by the aforementioned RDG and sign(*λ*_2_)*ρ* values.

Once F···HN^+^ hydrogen bonds are also expected to operate in **1**, the high energy difference between conformers in **2** relative to **1** should have another origin. Decomposition of the full energy in the conformers of **2** into Lewis and non-Lewis-type interactions, using the NBO method (*E* = *E*_L_ + *E*_NL_), shows that the *ag* conformer is more stabilized by hyperconjugation than *gg* in the gas phase (by 8.2 kcal mol^−1^), despite the large prevalence of the *gg* conformer (by 4.2 kcal mol^−1^) (Table 2). Such result indicates that *gg* is significantly favored by Lewis-type interactions, particularly by electrostatic effects (by ca*.* 12.4 kcal mol^−1^), in agreement with the electrostatic *gauche* effect. Even in implicit water, where the conformational energy is reduced to 1.3 kcal mol^−1^ and intramolecular dipolar effects are expected to be attenuated, the contribution from hyperconjugation for both conformers are very similar, while the electrostatic *gauche* effect favors the *gg* form by ca. 1.8 kcal mol^−1^.

Individual antiperiplanar hyperconjugative interactions similar to those responsible for the *gauche* effect in 1,2-difluoroethane favor the *gg* conformer in comparison with *ag*, but the sum of all electron delocalizations in **2** indicates that *ag* is more stabilized than *gg* by hyperconjugative effects (Table 2). Thus, the origin of the double *gauche* effect in **2** is predominantly due to electrostatic attraction between fluorines and the positively charged nitrogen, even in water solution, while hydrogen bond and hyperconjugation interactions play a secondary role for the conformational preference in **2**. This is different from findings for monofluorinated ethylammonium cations [6], and the results can be useful when evaluating the rules of stereochemical control during the development of multifluorinated alkylammonium cations.

## Conclusion

Both fluorine substituents bonded to a single carbon in an ethane fragment prefer the *gauche* orientation relative to an ammonium group, either in the gas phase or aqueous solution, giving rise to the so called double *gauche* effect. The origin of this effect in these media was found to be predominantly electrostatic, due to the attraction between the positively charged nitrogen and the electronegative fluorines, despite the participation of intramolecular hydrogen bond and hyperconjugation. These findings can be useful to predict the structure and stereochemistry of multifluorinated organic compounds with, e.g., pharmaceutical and/or agrochemical interest.

## Experimental

2,2-Difluoroethylamine hydrochloride (**2**) was purchased from Sigma-Aldrich and used without further treatment. The ^1^H NMR experiments were performed on a Bruker AVANCE III spectrometer operating at 499.87 MHz using ca. 20 mg mL^−1^ in D_2_O solution.

Compounds **1** and **2** present a total of 2 rotatable bonds, and considering the staggered conformations, as well as degenerate structures, five minima for **1** and two minima for **2** are expected. All geometries were optimized at the MP2/6-311++g(d,p) level [18–19] in the gas phase and using implicit solvent (H_2_O) according to the Polarizable Continuum Model (PCM) of Tomasi and coworkers [20]. Natural bond orbital (NBO) [21] analyses were also performed at the B3LYP/6-311++g(d,p) level of theory [19,22–23], including deletion of all antibonding and Rydberg-type orbitals. Spin–spin coupling constants were calculated at the BHandH/EPR-III level [24–25]. All these calculations were carried out using the Gaussian 09 program [26]. Quantum theory of atoms in molecules (QTAIM) calculations were performed to search for possible hydrogen bonds and their stabilities using the AIMAll program [27]. The non-covalent interaction (NCI) method was carried out by using the NCIPLOT program [28].

## Supporting Information

File 1 ^1^H NMR spectrum of **2**.

## References

[1] Lankin D C, Grunewald G L, Romero F A, Oren I Y, Snyder J P (2002). Org Lett.

[2] Snyder J P, Chandrakumar N S, Sato H, Lankin D C (2000). J Am Chem Soc.

[3] Lankin D C, Chandrakumar N S, Rao S N, Spangler D P, Snyder J P (1993). J Am Chem Soc.

[4] Sun A, Lankin D C, Hardcastle K, Snyder J P (2005). Chem–Eur J.

[5] Gooseman N E J, O'Hagan D, Peach M J G, Slawin A M Z, Tozer D J, Young J R (2007). Angew Chem, Int Ed.

[6] Silla J M, Silva W G D P, Cormanich R A, Rittner R, Tormena C F, Rittner R, Freitas M P (2014). J Phys Chem A.

[7] Goodman L, Gu H, Pophristic V (2005). J Phys Chem A.

[8] Buissoneaud D Y, van Mourik T, O'Hagan D (2010). Tetrahedron.

[9] Souza F R, Freitas M P, Rittner R (2008). J Mol Struct: THEOCHEM.

[10] O'Hagan D (2012). J Org Chem.

[11] Durig J R, Klaassen J J, Panikar S S, Darkhalil I D, Ganguly A, Guirgis G A (2011). J Mol Struct.

[12] Karplus M (1959). J Chem Phys.

[13] Karplus M (1960). J Phys Chem.

[14] Karplus M (1963). J Am Chem Soc.

[15] Johnson E R, Keinan S, Mori-Sánchez P, Contreras-García J, Cohen A J, Yang W (2010). J Am Chem Soc.

[16] Contreras-García J, Yang W, Johnson E R (2011). J Phys Chem A.

[17] Lane J R, Contreras-García J, Piquemal J-P, Miller B J, Kjaergaard H G (2013). J Chem Theory Comput.

[18] Head-Gordon M, Pople J A, Frisch M J (1988). Chem Phys Lett.

[19] Krishnan R, Binkley J S, Seeger R, Pople J A (1980). J Chem Phys.

[20] Tomasi J, Mennucci B, Cammi R (2005). Chem Rev.

[21] (2001). NBO.

[22] Becke A D (1988). Phys Rev A.

[23] Lee C, Yang W, Parr R G (1988). Phys Rev B.

[24] Becke A D (1993). J Chem Phys.

[25] Barone V, Chong D P (1996). Recent Advances in Density Functional Methods, Part I.

[26] (2009). Gaussian 09.

[27] (2013). AIMAll.

[28] Contreras-García J, Johnson E R, Keinan S, Chaudret R, Piquemal J-P, Beratan D N, Yang W (2011). J Chem Theory Comput.

