# Supplementary material for: Conformational analysis of 2,2-difluoroethylamine hydrochloride: double gauche effect
Source: Beilstein J Org Chem. 2014 Apr 16;10:877–82. doi: 10.3762/bjoc.10.84 (PMC3999868; doi:10.3762/bjoc.10.84)
Supplement: File 1 — 1H NMR spectrum of 2. [file Beilstein_J_Org_Chem-10-877-s001.pdf]

Supporting Information

for

Conformational analysis of 2,2-difluoroethylamine hydrochloride: double *gauche* effect

Josué M. Silla[1], Claudimar J. Duarte[2], Rodrigo A. Cormanich[2], Roberto Rittner[2], Matheus P. Freitas[1,*§]

[1]Department of Chemistry, Federal University of Lavras, P. O. Box 3037, 37200-000, Lavras, MG, Brazil and [2]Chemistry Institute, State University of Campinas, P. O. Box 6154, 13084-971, Campinas, SP, Brazil

Email: Matheus Puggina de Freitas[*] - matheus@dqi.ufla.br

[*]Corresponding author

[§]Tel.: +55 35 3829-1891; Fax: +55 35 3829-1271

^{1}H NMR spectrum of 2

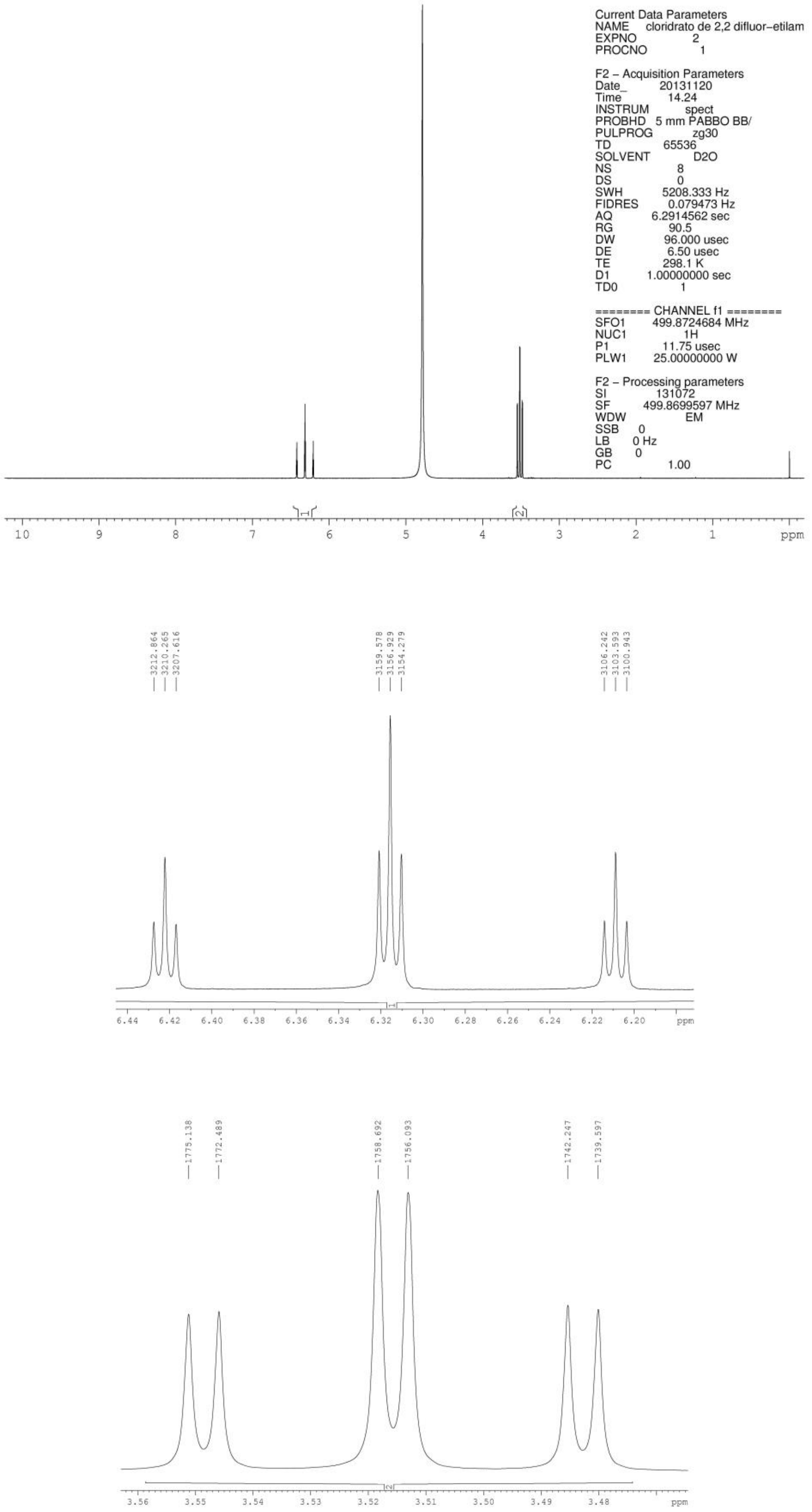

Figure S1. ^{1}H NMR spectrum of 2,2-difluoroethylamine hydrochloride in D_2O solution.
